# Mortality during treatment for tuberculosis; a review of surveillance data in a rural county in Kenya

**DOI:** 10.1371/journal.pone.0219191

**Published:** 2019-07-11

**Authors:** Osman A. Abdullahi, Moses M. Ngari, Deche Sanga, Geoffrey Katana, Annie Willetts

**Affiliations:** 1 Pwani University, Department of Public Health, Kilifi, Kenya; 2 KEMRI-Wellcome Trust Research Programme, Kilifi, Kenya; 3 Kilifi County TB Control Program, Kilifi, Kenya; 4 Kilifi County Department of Public Health, Kilifi, Kenya; University of Sheffield, UNITED KINGDOM

## Abstract

**Background:**

Globally in 2016, 1.7 million people died of Tuberculosis (TB). This study aimed to estimate all-cause mortality rate, identify features associated with mortality and describe trend in mortality rate from treatment initiation.

**Method:**

A 5-year (2012–2016) retrospective analysis of electronic TB surveillance data from Kilifi County, Kenya. The outcome was all-cause mortality within 180 days after starting TB treatment. The risk factors examined were demographic and clinical features at the time of starting anti-TB treatment. We performed survival analysis with time at risk defined from day of starting TB treatment to time of death, lost-to-follow-up or completing treatment. To account for ‘lost-to-follow-up’ we used competing risk analysis method to examine risk factors for all-cause mortality.

**Results:**

10,717 patients receiving TB treatment, median (IQR) age 33 (24–45) years were analyzed; 3,163 (30%) were HIV infected. Overall, 585 (5.5%) patients died; mortality rate of 12.2 (95% CI 11.3–13.3) deaths per 100 person-years (PY). Mortality rate increased from 7.8 (95% CI 6.4–9.5) in 2012 to 17.7 (95% CI 14.9–21.1) in 2016 per 100PY (P_trend_<0.0001). 449/585 (77%) of the deaths occurred within the first three months after starting TB treatment. The median time to death (IQR) declined from 87 (40–100) days in 2012 to 46 (18–83) days in 2016 (P_trend_ = 0·04). Mortality rate per 100PY was 7.3 (95% CI 6.5–7.8) and 23.1 (95% CI 20.8–25.7) among HIV-uninfected and HIV-infected patients respectively. Age, being a female, extrapulmonary TB, being undernourished, HIV infected and year of diagnosis were significantly associated with mortality.

**Conclusions:**

We found most deaths occurred within three months and an increasing mortality rate during the time under review among patients on TB treatment. Our results therefore warrant further investigation to explore host, disease or health system factors that may explain this trend.

## Introduction

Tuberculosis (TB) is the leading cause of death from a single infectious agent worldwide [1]. Global success of annual reduction in TB mortality by 3% is overshadowed by the estimated 1.7 million who died in 2016 of this curable disease, with the highest burden in sub-Saharan Africa [1, 2]. Hence an ambitious global target of reducing TB deaths by 95% from 2015 to 2035 has been set by the World Health Organization (WHO) [2].

Recent WHO estimates indicate most people with TB who die are no longer HIV-infected: 24% in 2016, a decline from 45% in 2008 [1]. The success of interventions in a policy environment focused on HIV can be attributed to access to integrated strategies for early TB diagnosis and treatment, including prioritising molecular diagnostics for these high risk populations [2]. Earlier reports on trends in risk factors for TB mortality since the recent decline in prevalence of people coinfected with HIV [3–6], a historical driver of TB [7], neglect inclusion of mortality rates, or report only on high-risk populations [8, 9]. These important reports are therefore limited to indicate the future direction of TB mortality or reflect a shift in the causal factors.

Several studies identify similar clinical and demographic characteristics among people with TB who die, primarily those with HIV coinfection without antiretroviral treatment [3, 8, 10, 11]. Other co-morbidities include older age, gender, being malnourished, concurrent diabetes mellitus, type of TB disease and antimicrobial resistance [3, 4, 8, 12, 13]. Thus, in the context of control measures against the HIV epidemic, the epidemiology of TB increasingly encompasses other high-risk populations [7].

Studies in sub-Saharan Africa potentially suggest the annual decline in TB mortality since 2000 [1] may be slowing down [14], or reversing [9], despite the sustained downward trend in new infections reported and people coinfected with HIV in the region. An urgent need exists to understand the dynamics of mortality after initiating treatment.

In this study, we aimed to estimate all-cause mortality rate, identify features associated with mortality, and describe mortality and survival trends during a five-year (2012–2016) period amongst patients during the six months of TB treatment period in Kilifi County, Kenya.

## Method

### Setting

Kilifi County is located on the coast of Kenya. The estimated total population is 1.4 million (national: 43 million) with an urban population at 26% [15]. The HIV prevalence is estimated as 3.7% (national 6%) and BCG immunization coverage 86% in 2016 [16]. The 2016 TB prevalence survey estimated 122/100,000 cases in Kilifi (compared to 170/100,000 nationally) and TB and HIV comorbidity at 17% [17]. Among notified TB cases a decline was observed in people coinfected with HIV at 31% in 2016 from 45% in 2008 [17, 18]. The GeneXpert Mycobacterium tuberculosis (MTB)/Rifampicin (RIF) (Cepheid USA) 4-module rapid molecular system was placed in two public hospitals in Kilifi during the study period (2012 and 2016) and reported 8–15 cases of resistance to TB treatment [17] within the study period. The main economic activity is subsistence agriculture [15].

In Kenya, all public health facilities offer TB treatment services. Health facilities send sputum for presumptive TB cases to the nearest TB diagnostic laboratory for GeneXpert MTB/RIF Cepheid or smear microscopy test with results expected to return to their respective health centers within 48 hours [19]. All TB patients are offered HIV counseling and testing after starting TB treatment. All HIV coinfected patients are linked to anti-retroviral treatment (ART) initiation and provision of HIV/AIDS care and support at the health facility, or the nearest site with services. According to national guidelines, ART is started at least two weeks after the start of TB therapy regardless of the CD4 cell count status. However, for severely immunosuppressed patients (those with CD4 <50 cells/uL), ART is initiated within the first two weeks of initiating TB treatment. People with HIV are given cotrimoxazole prophylaxis therapy (CPT) together with anti-TB treatment, provided there is no contraindication.

### Study design

A retrospective analysis of patients receiving anti-TB treatment in Kilifi County, Kenya. The primary outcome was all-cause mortality within 180 days after starting TB treatment. The risk factors examined were demographic and clinical features collected at the time of starting TB treatment.

### Study population

The study population consisted of persons with presumptive TB who started anti-TB treatment within the seven sub-county health facilities (Ganze, Kaloleni, Kilifi South, Kilifi North, Rabai, Malindi and Magarini) from January 2012 to December 2016, regardless of age.

### Data collection

All patients diagnosed with TB are registered in the electronic surveillance database of Kilifi County TB program (TIBU) by the sub-county coordinators within a month of starting treatment. TB was diagnosed by either direct smear microscopy, GeneXpert MTB/RIF for sputum, chest X-ray or using clinical symptoms according to WHO guidelines [20]. In hospitals with the capacity to use GeneXpert MTB/RIF and where smear microscopy was offered to the patient first, GeneXpert MTB/RIF was usually performed for patients who tested negative for smear microscopy.

Patients attended health facilities monthly for scheduled visits to collect their drugs. The Kilifi County TB program lack resources to conduct systematic community-treated patient follow-up at home. TB patient who failed to attend two consecutive scheduled monthly visits were traced through the community-based health workers and those not traceable were considered ‘lost-to-follow-up’. Patients moving out of Kilifi County advised to continue monthly clinics near to their new home and classified as ‘transferred’. Reported deaths were confirmed by a community home visit or from hospital records for those who died in hospital. For this analysis, outcomes during six months of follow-up were considered. For the `lost-to-follow-up’ and those `not evaluated’ patients, vital status at their last visit was used.

### Data and measurements

We categorized age into three preconceived groups;<15, 15 to 45, ≥45 years and used the ≥45 years as the reference because it had the highest mortality. The body mass Index was computed as weight (kgs) divided by the square of height in metres for adults aged ≥19 years. Children less than five years and 5 to 19 years old weight-for-height z-score (WHZ) were computed using the WHO 2006 and 2007 children growth references [21]. A nutritional status score was computed by combining the BMI and WHZ as follows; a) undernourished if WHZ<-2 or BMI<18.5, b) normal if WHZ -2 to +2 or BMI 18.5 to 25 and c) overweight if WHZ≥+2 or BMI≥ 25[22]. The TB patients were categorised as: Pulmonary vs. Extra-pulmonary and new vs. Re-treated. TB treatment outcomes were classified as ‘treatment completed’, `died’ or ‘lost-to-follow-up’ or ‘not evaluated’. Because this was surveillance data, smear microscopy test was not systematically performed at month five and therefore the ‘treatment completed’ included both cured and uncured patients who completed six months treatment. HIV was classified as: ‘HIV uninfected’, ‘HIV infected on ART’, ‘HIV infected not on ART’ and ‘unknown HIV status’. CD4 and viral load for HIV infected patients were not available. Anti-TB drug resistant data were not available.

### Statistical analysis

All the statistical analysis was performed using Stata version 15.1 (StataCorp, College Station, TX, USA). For patients missing specific variable data, such as nutrition or HIV status, a separate missing category was allocated (unknown/missing) and analyzed, because data were assumed not to be missing at random.

We did a single event survival analysis since date of starting TB treatment. Follow-up time was from the date of starting anti-TB treatment to a maximum of 180 days or time of death/lost to follow-up/date last seen. Mortality rates were computed as the number of deaths per 100person-year (PY). Yearly trend all-cause mortality rate within 180 days after TB treatment initiation was tested using an extension of Wilcoxon rank-sum test for trend across ordered groups [23].

We plotted smoothed cumulative subhazard curves using the STATA *stcurve* command and compared survival distributions between those alive and died using log-rank test. Since lost to follow-up or outcome ‘not evaluated’ would preclude the probability of observing death (our outcome of interest), we treated these two events as competing events. We thus used Fine and Gray competing risk analysis method instead of the conservative cox-regression models to examine putative risk factors for all-cause mortality and reported the effects using sub-distribution hazards ratios (SHR) and their respective 95% confidence intervals [24]. Briefly, the competing risk analysis calculates the cumulative hazards of death associated with the exposure of interest while adjusting for other covariates and accounts for the competing events, rather than censoring these events as is the case with cox-regression models.

In the multivariable competing risk analysis model, we used backwards stepwise approach to retain independent variables with P<0.1 and reported adjusted SHR for variables in the final model with P<0.05. We performed internal validation of the multivariable model by computing bootstrapped area under receiver operating curve (AUC) estimated by using probit model resampled 100 times with replacement. To account for the clustering of study patients within sub-counties, we performed both univariate and multivariable competing risk models with random effects intercept allowing for sub-counties clustering. To test heterogeneity of mortality rates across the seven sub-counties, we tested the hypothesis that the mortality rates were not different using meta-analysis method with random effects.

We also performed a sub-analysis to examine risk of death between persons with presumptive TB with a confirmed TB diagnosis using either positive sputum microscopy or GeneXpert MTB/RIF and those with clinical TB symptoms (including extra pulmonary TB).

### Ethical considerations

Ethical approval was obtained from Pwani University Ethical Review Board (ERC/PU-STAFF/005/2018) and permission granted by the Kilifi County Ethical Research Committee to access the anonymized data.

## Results

### Baseline characteristics

**Fig 1:**
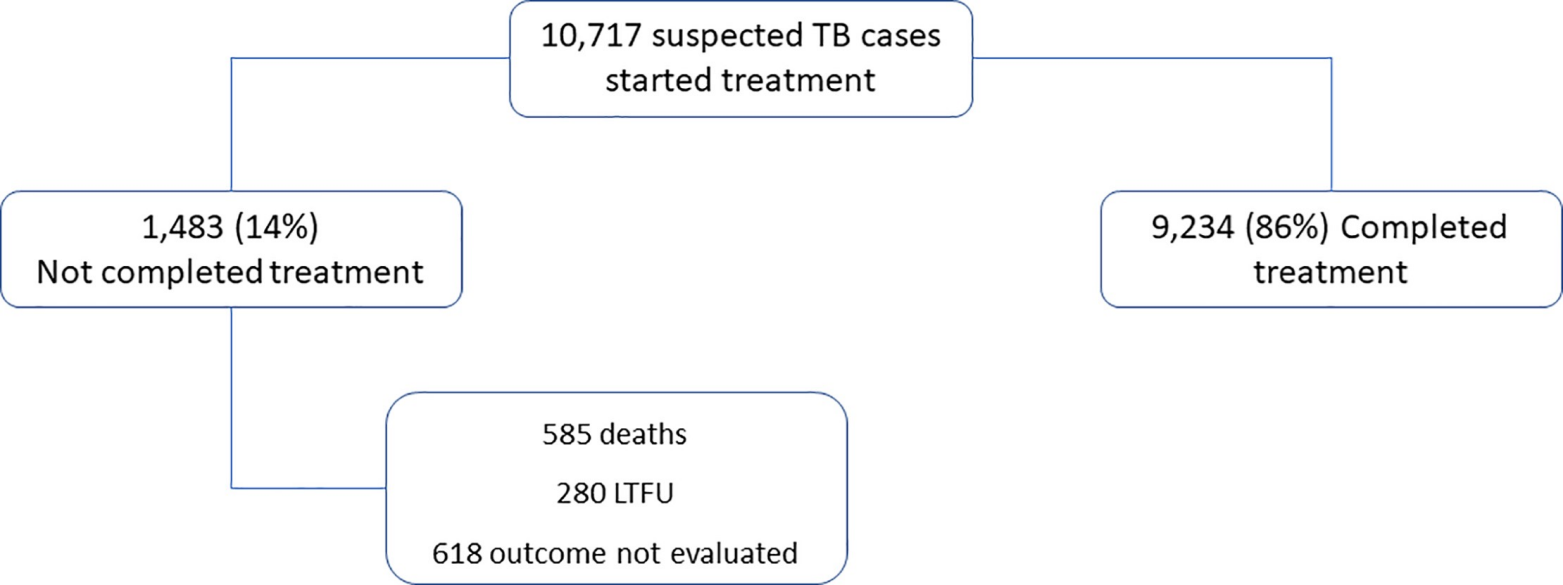
Flow of TB cases registered in the TIBU programme (2012 to 2016), Kilifi County. LTFU-Lost to follow-up.

From 2012 to 2016, 10,717 patients were started on TB treatment in 121 health facilities in Kilifi County (**Fig 1**). The patients’ range and median (IQR) age was 1 to 84 and 33 (24–45) years respectively and 4,529 (42%) were female. 3163 (30%) patients were HIV-infected; of which 2963 (94%) on ART while 3134 (99%) receiving cotrimoxazole prophylaxis. Approximately one third of the participants 3,694 (34%)had normal nutrition status, whilst 4,847 (45%)undernourished, mostly from Kaloleni and Rabai sub-counties. 832 (7.8%)found to be overweight or obese (**Table 1 and S1 Fig**).

**Table 1:** Characteristics of the TB patients at the time of initiating TB treatment in Kilifi County, Kenya.

Features	All patients(N = 10,717)	Completed treatment (N = 9,234)	Died (N = 585)	LTFU^1^ (N = 280)	Outcome not evaluated (N = 618)
Age in years					
<15 years	1,256 (12)	1,166 (93)	33 (2.6)	19 (1.5)	38 (3.0)
15 to 45 years	6,598 (61)	5,688 (86)	274 (4.2)	196 (3.0)	440 (6.7)
45 and above years	2,863 (27)	2,380 (83)	278 (9.7)	65 (2.3)	140 (4.9)
Sex					
Male	6,188 (58)	5,260 (85)	338 (5.5)	198 (3.2)	392 (6.3)
Female	4,529 (42)	3,974 (88)	247 (5.5)	82 (1.8)	226 (5.0)
Patient type					
New cases	9,708 (91)	8,416 (87)	512 (5.3)	237 (2.4)	543 (5.6)
Re-treatment cases	1,009 (9.4)	818 (81)	73 (7.2)	43 (4.3)	75 (7.4)
TB type					
Pulmonary	9,413 (88)	8,037 (85)	521 (5.5)	259 (2.8)	596 (6.3)
Extrapulmonary	1,304 (12)	1,105 (85)	99 (7.6)	31 (2.4)	69 (5.3)
Type of health facility					
Public	8,361 (78)	7,247 (87)	427 (5.1)	202 (2.4)	485 (5.8)
Private	2,173 (20)	1,820 (84)	155 (7.1)	73 (3.4)	125 (5.8)
Prisons	183 (2.0)	167 (91)	3 (1.6)	5 (2.7)	8 (4.4)
DOT^2^					
Family-based	9,280 (87)	8,029 (87)	506 (5.5)	223 (2.4)	522 (5.6)
Community volunteer	800 (7.5)	688 (86)	33 (4.1)	36 (4.5)	43 (5.4)
Health worker	637 (5.9)	517 (81)	46 (7.2)	21 (3.3)	53 (8.3)
Nutrition status					
Undernourished	4,847 (45)	4,078 (84)	322 (6.6)	147 (3.0)	300 (6.2)
Normal	3,694 (34)	3,252 (88)	155 (4.2)	82 (2.2)	205 (5.6)
Overweight	832 (7.8)	703 (85)	45 (5.4)	12 (1.4)	72 (8.7)
Missing anthropometrics	1,344 (13)	1,109 (82)	98 (7.3)	49 (3.7)	88 (663)
HIV status					
HIV uninfected	7,413 (69)	6,556 (88)	251 (3.4)	190 (2.6)	416 (5.6)
HIV infected on ARVS	2,963 (28)	2,429 (82)	292 (9.9)	81 (2.7)	161 (5.4)
HIV infected not on ARVS	200 (1.9)	139 (70)	33 (17)	5 (2.5)	23 (12)
Unknown HIV status	141 (1.3)	110 (78)	9 (6.4)	4 (2.8)	18 (13)
Treatment regimen					
2RHZE/4RH	9,380 (88)	8,126 (87)	491 (5.2)	237 (2.5)	526 (5.6)
2SRHZE/1RHZE/5RHE	1,078 (10)	872 (81)	85 (7.9)	40 (3.7)	81 (7.5)
2RHZ/4RH	234 (2.2)	213 (91)	7 (3.0)	3 (1.3)	11 (4.7)
Others	25 (0.2)	23 (92)	2 (8.0)	0	0
Sub County					
Kilifi North	1,757 (16) ((16)1,717	1,450 (83)	113 (6.4)	74 (4.2)	120 (6.8)
Kilifi South	1,717 (16)	1,481 (86)	59 (3.4)	53 (3.1)	124 (7.2)
Kaloleni	2,049 (19)	1,727 (84)	165 (8.1)	38 (1.9)	119 (5.8)
Malindi	2,824 (26)	2,482 (88)	107 (3.8)	71 (2.5)	164 (5.8)
Magarini	1,284 (12)	1,160 (90)	49 (3.8)	22 (1.7)	53 (4.1)
Ganze	536 (5.0)	461 (86)	43 (8.0)	13 (2.4)	19 (3.5)
Rabai	550 (5.1)	473 (86)	49 (8.9)	9 (1.6)	19 (3.5)
Year of diagnosis					
2012	2,610 (24)	2,316 (89)	93 (3.6)	0	201 (7.7)
2013	2,274 (21)	1,972 (87)	115 (5.1)	8 (0.4)	179 (7.9)
2014	2,271 (21)	1,982 (87)	137 (6.0)	73 (3.2)	79 (3.5)
2015	1,873 (17)	1,591 (85)	110 (5.9)	101 (5.4)	71 (3.8)
2016	1,689 (16)	1,373 (81)	130 (7.7)	98 (5.8)	88 (5.2)

^1^LTFU-Lost to follow-u

^2^DOT-directly observed treatment; all results are N (%).

Of the 10,717 patients, 1,009 (9.4%) were re-treatment cases. Overall, 1,304 (12%) were extrapulmonary cases and 9,413 (88%) pulmonary TB cases. A total of 4,470/10,717 (42%) had positive sputum microscopy, 163/10,717 (1.5%) were diagnosed by GeneXpert and 4,780/10,717 (45%) were diagnosed by clinical signs only (**Table 1).**

**Fig 2:**
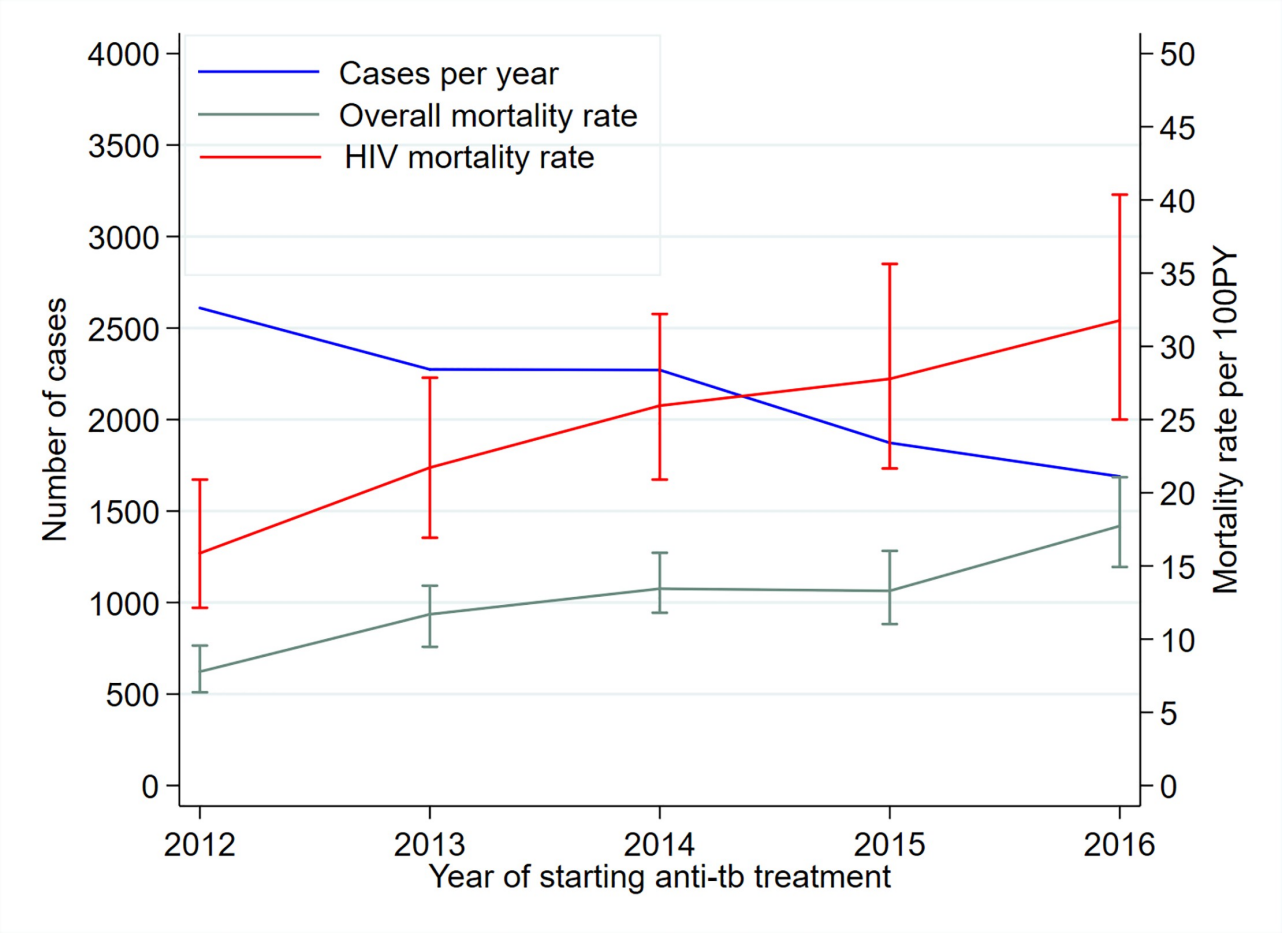
Changes in number of TB cases, overall mortality rate and HIV infected mortality rate per 100PY over the years. Linear trend P-values for number of cases (0·02-declining), overall mortality rate (<0·0001-increasing) and HIV mortality rate (0·001-increasing), PY-person years, the plotted overall and HIV curves are the mean and 95% CI mortality rates.

The number of patients starting anti-TB treatment declined from 2,610(24% of the cohort) in 2012 to 1,689(16%) in 2016 (P_trend_ = 0.02) (**Fig 2**). The number of study participants diagnosed for TB with GeneXpert increased across the five years (P_trend_ = 0.003) and those diagnosed through clinical signs declined (P_trend_ = 0.008) (**S1 Table**).

### All-cause mortality rate post TB initiation

During the six months follow-up, 9,234(86%) patients completed TB treatment and 1,483 (14%) did not complete treatment. Overall, 585 (5.5%) died, 280 (2.6%) were lost to follow-up and 618(5.8%) outcome not evaluated (**Fig 1**). In total 898/10,717 (8.4%) of the patients found either lost-to-follow-up or their outcome not evaluated and included in the regression model as competing events. **Table 1** shows characteristics of participants by their treatment outcome.

**Fig 3:**
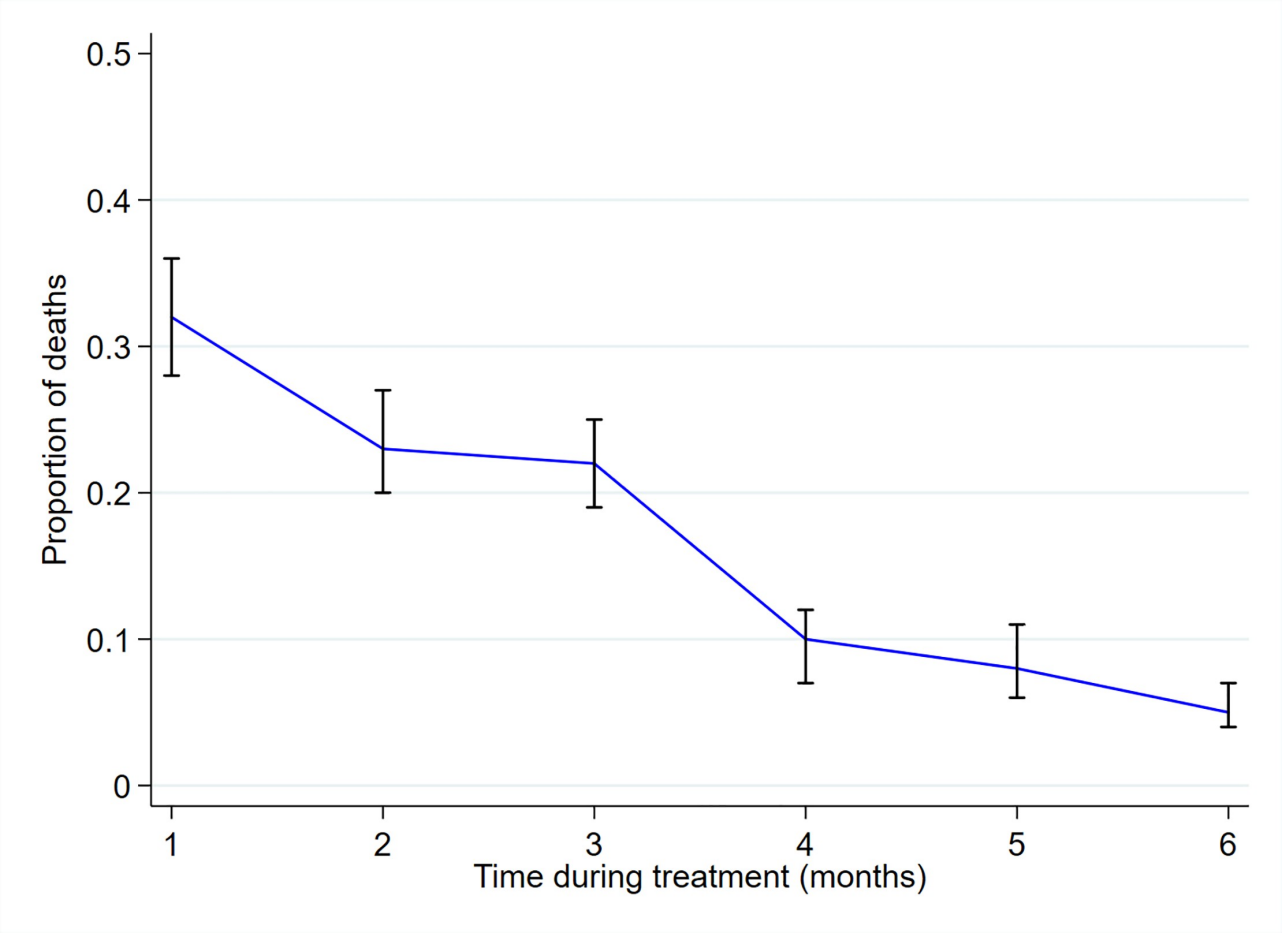
Monthly proportion of deaths after starting TB treatment. The plotted curves are the monthly proportion of deaths (deaths in each month divided by total deaths) and 95% Confidence Intervals.

Patients were in follow-up for 5175.5PY; mortality rate of 12.2 (95% CI 11.3–13.3) per 100PY. The mortality rate increased from 7.8 (95% CI 6.4–9.5) in 2012 to 17.7 (95% CI 14.9–21.1) in 2016 per 100PY (P_trend_<0.0001) (**Fig 2 and S1 Table**). 449/585 (77%) of all the deaths occurred within the first three months after starting TB treatment; mortality rate 314.0 (95% CI 286.2–344.4) deaths per 100PY. Overall, the monthly mortality case ratio declined from 32% (95% CI 28 to 36) during the first month of treatment to 5% (95% CI 4 to 7) between fifth and sixth month of treatment (P_trend_<0.001) **Fig 3**. Among the early deaths in the first three months of treatment, mortality rate increased from 175.8 (95% CI 138.1–223.7) in 2012 to 351.0 (95% CI 290.4–424.2) deaths per 100PY in 2016(P_trend_0.03) **S2 Fig**. However, among the 136/585 (23%) deaths occurring after 3 months of treatment, there was no evidence of linear increase in mortality; from 2.33 (95% CI 1.60–3.40) in 2012 to 3.27 (95% CI 2.17–4.93) deaths per 100PY in 2016 (P_trend_ = 0.43) **S2 Fig**). The mortality rate was heterogeneous across the seven sub-counties with Kilifi south having the lowest rate 7.6 (95% CI 5.9–9.8) and Rabai sub-county with the highest rate of 20.3 (95% CI 15.3–26.8) per 100PY; I^2^ = 92.1%, P<0.0001 (**S3 Fig**). Among HIV infected patients, the mortality rate increased from 15.9 (95% CI 12.1–20.8) in 2012 to 31.8 (95% 25.0–40.4) per 100PYin 2016 (P_trend_ = 0.001) (**Fig 2 and S1 Table**). Median time to death (IQR) declined from 87 (40–100) days in 2012 to 46 (18–83) days in 2016 (P_trend_ = 0.04) (**S1 Table).**

### Risk factors for all-cause mortality post-TB treatment initiation

**Fig 4:**
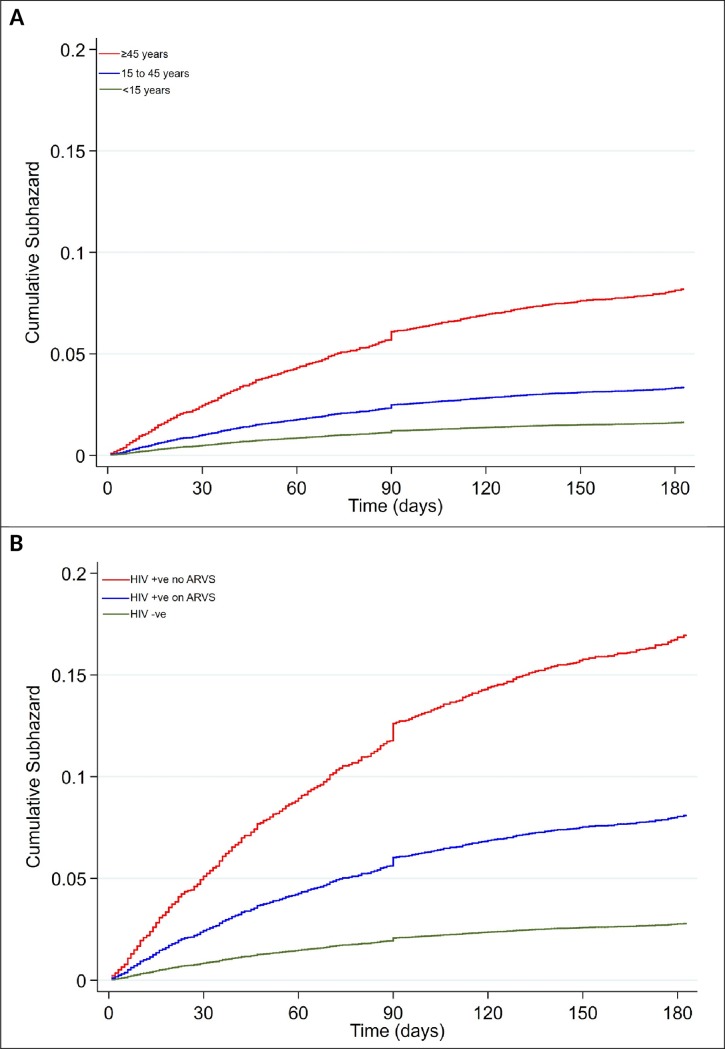
Kaplan-meier graphs for all-cause mortality by: A-age group and B-HIV status.

Age (age<45 years was protective compared to ≥45 years), female sex, extrapulmonary TB, low nutritional status, HIV infection, 2SRHZE/1RHZE/5RHE treatment regimen and year of diagnosis were independent risk factors for all-cause mortality within 180 days after initiation of TB treatment (**S2 Table, Fig 4A and 4B and Table 2**). There were 251/7413 (3.4%) deaths among the HIV uninfected people: mortality rate 7.3 (95% CI 6.5–7.8) per 100PY and 325/3163 (10.3%) deaths among the HIV infected people: mortality rate 23.1 (95% CI 20.8–25.7) per 100PY. Overall, HIV infection was associated with mortality, adjusted SHR: 3.26 (95% CI 2.76–3.86). Among the HIV infected patients, 33/130 (25%) died amongst those not on ARVS and 292/1,463 (20%) amongst those on ARVS. Being on ARVS was associated with 43% reduction in risk of death (crude SHR 0.57 (95% CI 0.37–0.87)) compared to not being on ARVS among the HIV infected patients. There was no significant difference in time to death between patients on ARVS and not on ARVS (P = 0.08), however those not on ARVS were in follow-up for a shorter period (mean of 142 days) compared to those on ARVS (mean of 159 days) P<0.0001) amongst HIV infected patients.

**Table 2:** Risk factors for all-cause mortality 180 days after initiation of TB treatment in Kilifi County, Kenya.

Features	Deaths (N = 585)	Mortality rate per 100PY (95% CI)	Adjusted SHR (95% CI)	P-value
Age in years				
<15 years	33 (2.6)	5.7 (3.5–8.8)	0.26 (0.22–0.32)	<0.0001
15 to 45 years	274 (4.2)	9.3 (6.8–12.7)	0.38 (0.34–0.41)	<0.0001
45 and above years	278 (9.7)	22.3 (16.5–30.8)	Reference	
Sex				
Male	338 (5.5)	12.3 (8.6–17.6)	Reference	
Female	247 (5.5)	12.1 (8.7–17.2)	0.86 (0.80–0.93)	<0.0001
TB type				
Pulmonary	491 (5.2)	11.7 (8.2–16.6)	Reference	
Extrapulmonary	94 (7.2)	16.4 (11.5–24.0)	1.22 (1.01–1.48)	0.04
Type of health facility				
Public	427 (5.1)	11.4 (8.5–15.1)	Reference	
Private	155 (7.1)	16.3 (7.3–48.8)	1.24 (0.83–1.85)	0.28
Prison	3 (1.6)	3.6 (2.7–5.3)	0.33 (0.27–0.42)	<0.0001
Nutrition status				
Undernourished	305 (6.3)	14.20 (12.68,15.88)	1.46 (1.39–1.53)	<0.0001
Normal	151 (4.1)	8.97 (7.65, 10.52)	Reference	
Overweight	41 (4.9)	11.09 (8.17, 15.07)	1.06 (0.78–1.46)	0.70
Missing anthropometrics	88 (6.6)	15.07 (12.23,18.58) 18.58)	1.64 (1.07–2.52)	0.02
HIV status				
HIV uninfected	251 (3.4)	7.5 (4.8–11.6)	Reference	
HIV infected on ARVS	292 (9.9)	22.7 (17.5–29.5)	3.06 (2.38–3.94)	<0.0001
HIV infected not on ARVS	33 (17)	42.4 (31.9–54.4)	6.12 (3.15–11.89)	<0.0001
Unknown HIV status	9 (6.4)	15.7 (6.2–54.1)	1.91 (0.99–3.68)	0.05
Treatment regimen				
2RHZE/4RH	491 (5.2)	11.7 (8.2–16.7)	Reference	
2SRHZE/1RHZE/5RHE	85 (7.9)	18.1 (13.8–24.4)	1.24 (1.05–1.46)	0.01
2RHZ/4RH	7 (3.0)	6.6 (2.8–14.1)	0.89 (0.49–1.61)	0.70
Others	2 (8.0)	16.6 (5.1–25.6)	1.41 (0.50–4.03)	0.52
Year of diagnosis				
2012	93 (3.6)	7.8 (4.3–14.0)	Reference	
2013	115 (5.1)	11.4 (6.8–20.1)	1.32 (1.05–1.67)	0.02
2014	137 (6.0)	13.5 (9.0–20.5)	1.61 (1.25–2.08)	<0.0001
2015	110 (5.9)	13.3 (10.0–18.0)	1.69 (1.14–2.50)	0.009
2016	130 (7.7)	17.7 (14.4–21.8)	2.43 (1.50–3.93)	<0.0001
Model Performance				
AUC (95% CI)			0.75 (0.73–0.76)	
Bootstrapped AUC (95% CI)			0.74 (0.73–0.76)	

SHR-Sub-distribution hazard ratios, DOT-directly observed treatment, PY-person year, AUC-Area under receiver operating curve, The SHR are obtained using Fine and Gray competing risk regression analysis.

In the sub-analysis, 4,633(43%) were bacteriologically confirmed TB cases, while 6,084 (57%) were clinically diagnosed. Amongst confirmed TB cases, 169/4,633 (3.7%) died while 416/6,084 (6.8%) died among the clinically diagnosed (P<0.0001). Among confirmed TB cases, we observed a similar increase in mortality rate trend to the whole cohort; an increase from 5.7 (95% CI 3.8–8.4) deaths per 100PY in 2012 to13.2 (95% CI 10.1–17.2) deaths per 100PY in 2016(P_trend_ = 0.007) (**S3 Table**). Compared to the clinically diagnosed patients, confirmed TB cases had a lower risk of dying; age, HIV and sex adjusted SHR 0.56 (95% CI 0.40–0.79). Factors associated with mortality amongst the 43% confirmed TB cases were similar to those of the overall cohort (**S3 Table**). Old age, extrapulmonary TB and HIV infection were associated with both deaths within and after three months of starting TB treatment (**S4 Table).**

## Discussion

Our results provide evidence of an increase in the risk of dying and shortened time to death among patients on anti-TB treatment during a period of declining number of TB cases. This increase is driven primarily by individuals coinfected with HIV, including in the subgroup of confirmed TB cases. However, HIV infected patients on ARVs had lower risk of mortality compared to those not on treatment, as found in previous studies [25]. Earlier studies in the region, including Kenya, report a decline in both the number of TB cases and mortality, however defined by case fatality ratios [4, 5]. This study found more than three quarters of the deaths occurred within three months of starting TB treatment. These early deaths had an increasing mortality rate whilst deaths occurring after the third month of TB treatment remained fairly constant from 2012 to 2016. Undetected anti-TB resistance, poor adherence to treatment, late TB diagnosis and treatment delay observed in TB endemic countries may contribute to this increasing mortality trend [26–28].

The trend of most deaths occurring early suggests a late TB diagnosis or delay in starting treatment, including ARVS amongst HIV infected patients. More than half of TB cases were diagnosed using clinical signs suggesting using molecular tools to diagnosis TB proved challenging in the county. Clinical diagnosis conducted when symptoms are visible to the clinician potentially at a more advanced stage of TB disease. It is therefore not surprising most deaths occurred early. The policy of testing TB and having the patients receive their results after 48 hours necessarily delays starting of treatment [19]. Although we do not know the proportion of the patients who did not know their HIV status before the TB diagnosis, it is possible most of them started taking ARVS after TB treatment and therefore this provided a window for early deaths. We suspected an increasing trend in delay of HIV diagnosis and starting ARVs especially amongst the elderly as a result of an HIV testing campaign biased towards the youths in Kenya [29].

Our study found being immunosuppressed presented a higher risk for dying during TB treatment. As documented in other studies, people of older age, malnourished (low and high BMI), and HIV coinfected are high risk groups [3, 4, 10, 12]. Patients missing BMI/z-score had elevated risk of mortality suggesting data were not missing at random and the need to be included in mortality estimates. The WHO estimates show an increase in diabetes among Kenyans, independently associated with higher TB mortality in other studies [1, 13]. The higher burden of non-communicable diseases among older Kenyan adults potentially increases mortality during TB treatment [30].

The explanation for the observed decline in survival time from starting treatment to death is unclear. All major clinical and demographic factors remained unchanged during the 5-year study period, including HIV coinfection at 27–30%. In 2012, Kilifi County started implementing the new WHO screening and testing algorithms and molecular technology for TB and drug resistance testing (GeneXpert MTB/Rif Cepheid USA) [17]. This context of heightened TB awareness may explain the decline in notified TB cases as clinical diagnosis of persons with presumptive TB reduced. The Kilifi County has recorded annual population growth of 3.1% suggesting the declining notified TB cases has no correlation with the population changes. Linking the GeneXpert drug resistance data with TIBU surveillance information potentially creates an opportunity to explain mortality trends in this type of TB.

This study identified only a brief window to intervene in the TB pathway from starting treatment to death. As reported in other studies, more than half of the deaths occur within the 2-month of intensive treatment phase [3, 12, 31]. Some authors attribute the short survival time to delays in TB diagnosis and/or initiation of anti-TB and antiretroviral treatment, severe disease, undiagnosed drug-resistant TB or co-morbidities, low adherence to anti-TB drugs and challenges in health care access [3, 32, 33]. The heterogeneity between sub-counties in mortality rates and BMI observed in this study may be explained by inequities and inadequate access to TB services across the county. Of value to the TB program is the finding that current TB, ARVS and cotrimoxazole strategies alone may not be optimal in reducing the risk of mortality, as found in other studies [11, 25]. Notably, the protective effect of ARV therapy was less (43%) in our study than in the meta-analysis conducted by Odone et al, 2014, despite 94% ARV coverage in this cohort [34, 35].

The major strength of this study was its large size, systematic data collection process and comprehensiveness of the data available for this analysis within the five-year period. Our established collaboration with the sub-county program coordinators allowed verification of inconsistencies and incompleteness in the data at facility level.

Our study is limited by several factors. TB diagnosis using the WHO clinical signs are typical in resource-limited settings however was necessarily subjective. A sub-analysis found confirmed TB cases have a lower mortality risk than clinically diagnosed patients but identified similar increasing mortality rate trend and risk factors of dying suggesting the clinical diagnosis identifies patients most at risk of death. We could not ascertain the vital status of the 898/10,717 (8.4%) patients not completing treatment due either lost-to-follow-up (LTFU) or outcome not evaluated. To account for this limitation, we treated these events (LTFU/outcome not evaluated) as competing events with the outcome (mortality) in the analysis. The higher mortality amongst adults above 45 years is potentially attributed to other old-age related illness, including non-communicable diseases (heart related or cancer), low CD4 counts or drug resistance which are not routinely collected for the TIBU database. Drug resistance surveillance systems report low incidence at County and National level [17]. Programmatic surveillance data potentially underestimates or overestimates TB mortality, for example it did not allow us to examine the causes of community all-cause mortality and undiagnosed TB. Reliance on verbal autopsy and missed TB cases underestimates the true burden of TB related mortality. These limitations are inherent in programmatic data and difficult to prevent during the analysis.

Future research is needed to better understand the dynamics of deaths among TB patients on treatment. The role of factors that delay TB diagnosis and treatment require exploring, providing vital information to inform early diagnosis and avert most of the TB attributable deaths. HIV co-infection was the main factor associated with mortality, however little is known when the HIV diagnosis was made, ARVs started and monitoring of changing CD4 counts and viral load while on treatment. The effect of systematically screening for HIV at time of starting TB treatment and either linking those infected with HIV clinics or promptly starting ARVS should be evaluated. The role of anti-TB drug resistant, adherence to the TB treatment, health system bottlenecks and other social poverty-related constrains need further exploration.

## Conclusions

We found most deaths occurred within first three months and an increasing mortality rate among patients on TB treatment. Our findings warrant further investigation to go beyond already established indicators to explore host, disease or health system factors that may explain the observed trend.

## Supporting information

S1 Fig Proportions of different levels of nutritional status from the seven sub counties. (TIFF)

S2 Fig A- Annually mortality rate per 100PY for deaths occurring within three months of starting TB treatment and B- Annually mortality rate per 100PY for deaths occurring after three months of starting TB treatment.
(TIFF)

S3 Fig Mortality rates per 100 person-years from the seven sub counties. (TIFF)

S1 Table Changes of selected participants’ characteristics and outcomes across the years. (DOCX)

S2 Table Univariable associations between features at initiating TB treatment and deaths within six months of follow up. (DOCX)

S3 Table Associations between features at initiating TB treatment and deaths within six months of follow-up among confirmed TB cases. (DOCX)

S4 Table Associations between features at initiating TB treatment and deaths stratified by deaths within and after three months of starting TB treatment. (DOCX)

## Abbreviations

TB: Tuberculosis
WHO: World Health Organization
HIV: Human immunodeficiency virus
ART: antiretroviral therapy
BCG: Bacillus Calmette–Guérin
CPT: cotrimoxazole prophylaxis therapy
