# Supplementary figures and images for: Mortality during treatment for tuberculosis; a review of surveillance data in a rural county in Kenya
Source: PLoS One. 2019 Jul 11;14(7):e0219191. doi: 10.1371/journal.pone.0219191 (PMC6622488; doi:10.1371/journal.pone.0219191)

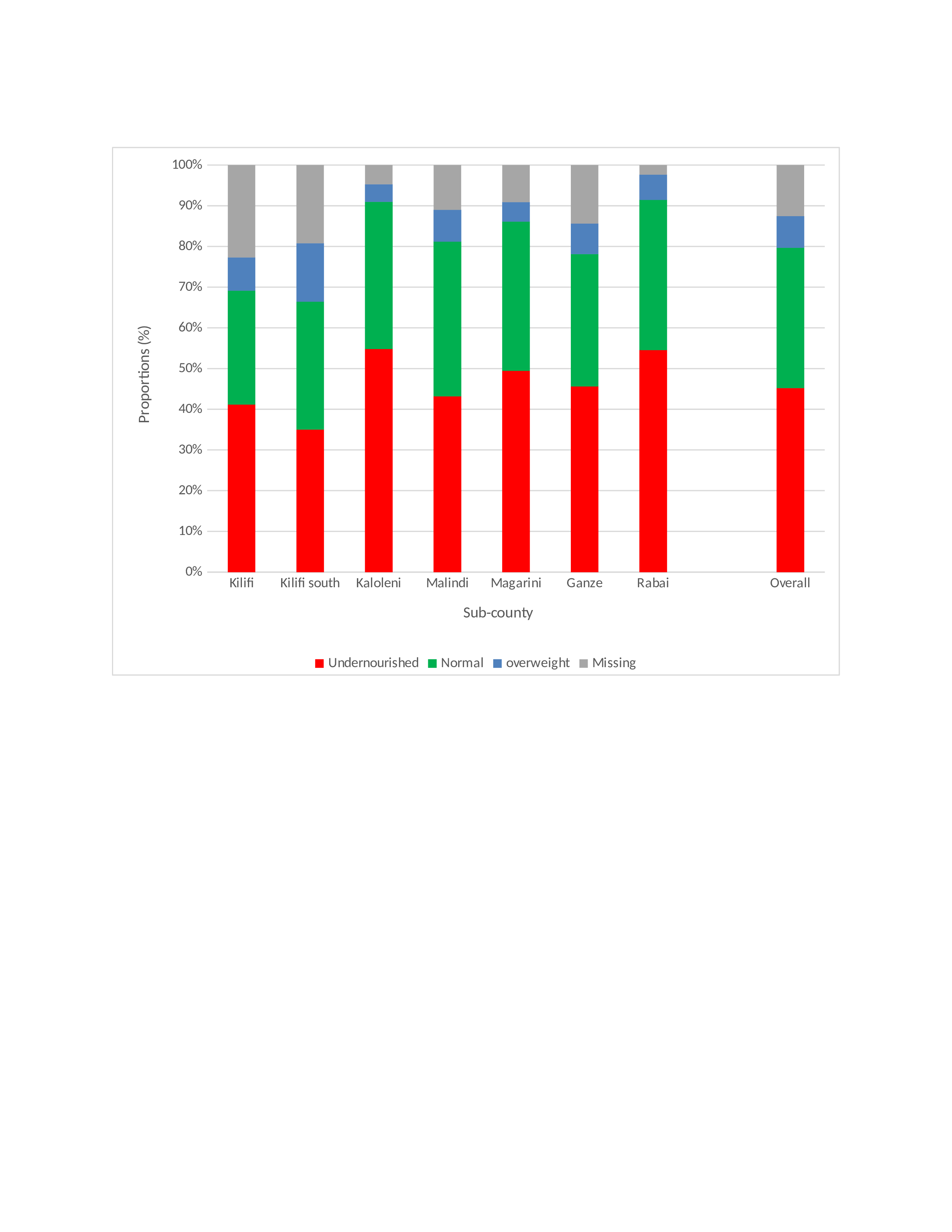

Supplement: S1 Fig — (TIFF) [file pone.0219191.s001.tiff]

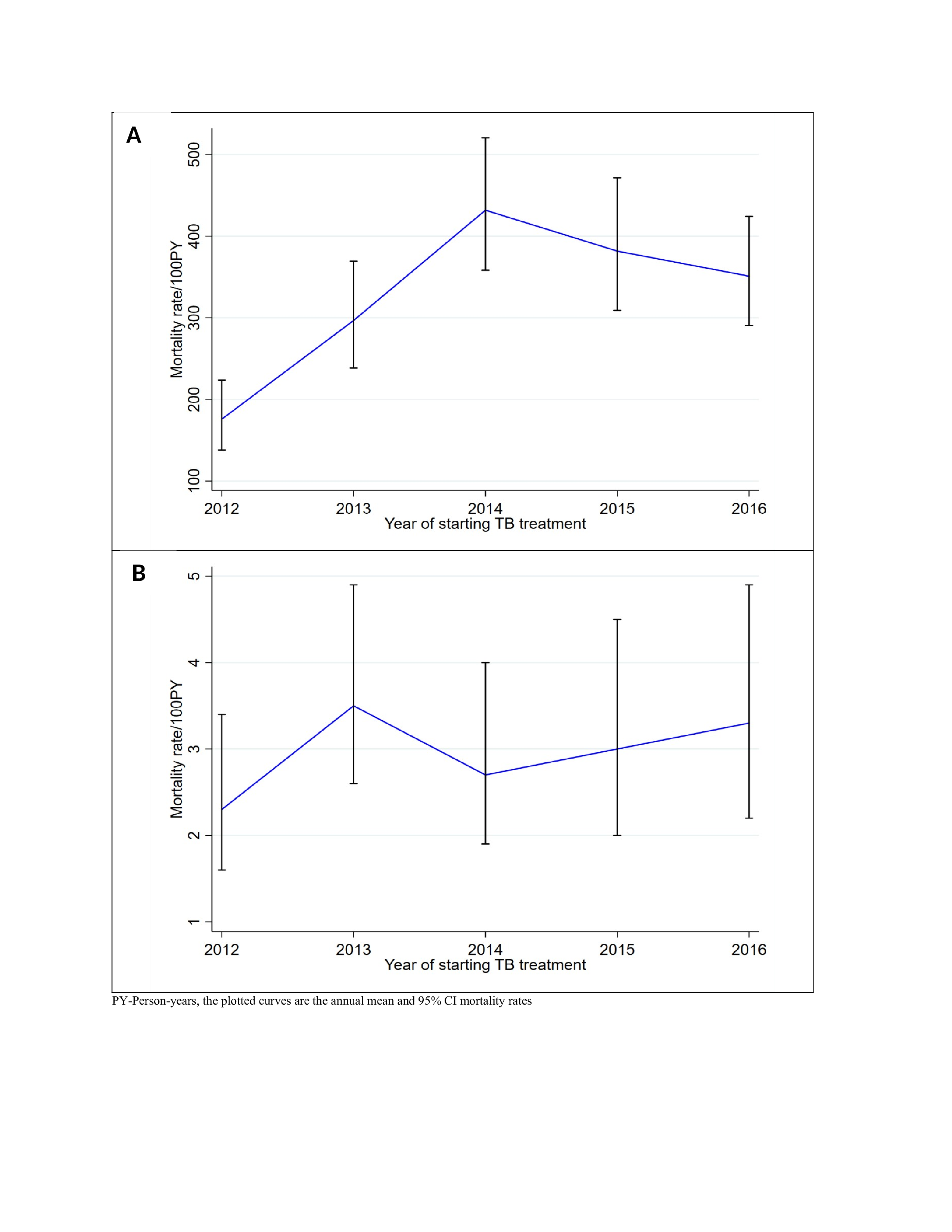

Supplement: S2 Fig — A- Annually mortality rate per 100PY for deaths occurring within three months of starting TB treatment and B- Annually mortality rate per 100PY for deaths occurring after three months of starting TB treatment. (TIFF) [file pone.0219191.s002.tiff]

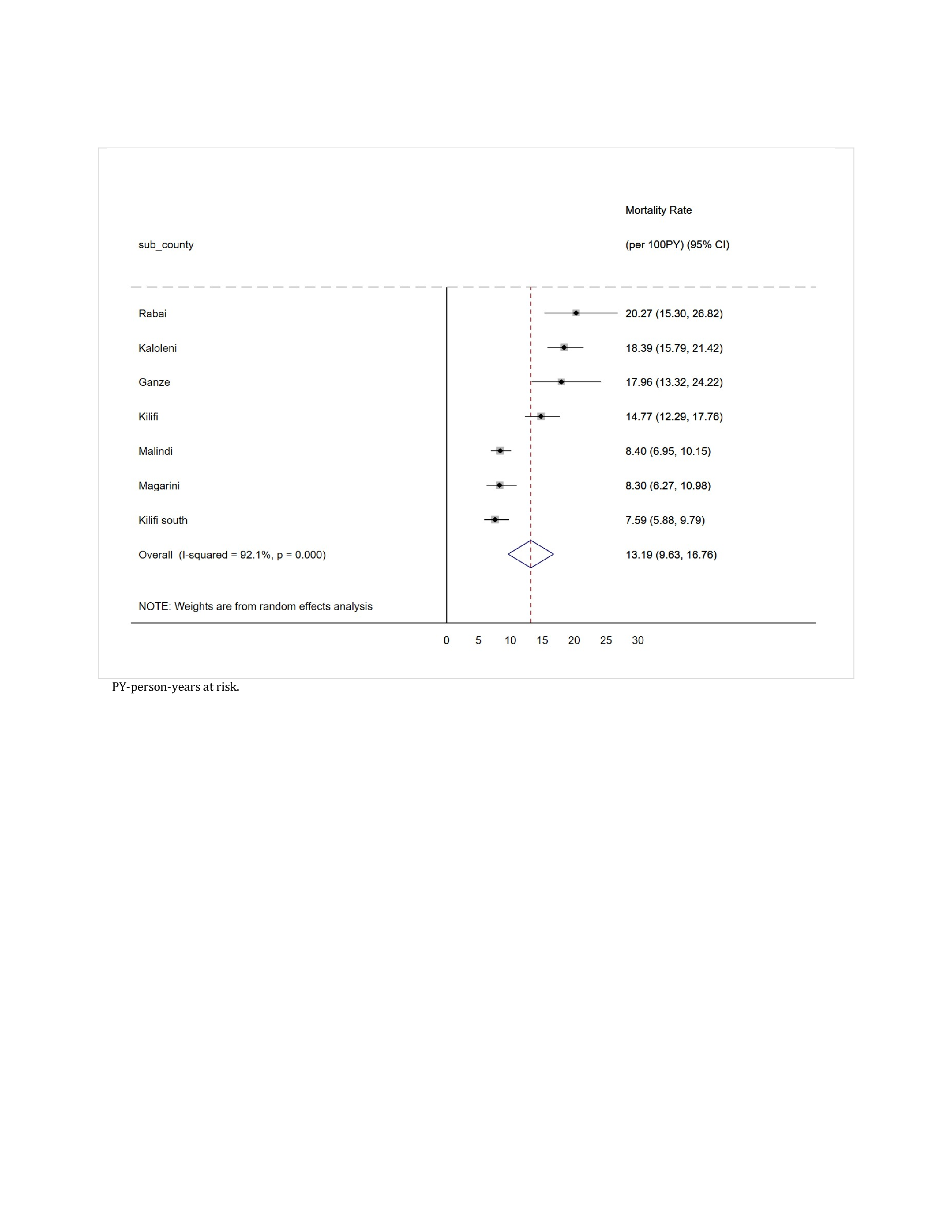

Supplement: S3 Fig — (TIFF) [file pone.0219191.s003.tiff]
