# Supplementary material for: Mortality during treatment for tuberculosis; a review of surveillance data in a rural county in Kenya
Source: PLoS One. 2019 Jul 11;14(7):e0219191. doi: 10.1371/journal.pone.0219191 (PMC6622488; doi:10.1371/journal.pone.0219191)
Supplement: S1 Table — (DOCX) [file pone.0219191.s004.docx]

**S1 Table**: Changes of selected participants’ characteristics and outcomes across the years.

| Features | 2012  N=2,610 | 2013  N=2,274 | 2014  N=2,271 | 2015  N=1,873 | 2016  N=1,689 | Trend P-value |
| --- | --- | --- | --- | --- | --- | --- |
| Mortality |  |  |  |  |  |  |
| Overall case fatality | 93 (3.6) | 115 (5.1) | 137 (6.0) | 110 (5.9) | 130 (7.7) | <0.0001 |
| Mortality rate per 100PY | 7.8 | 11.4 | 13.5 | 13.3 | 17.7 | <0.0001 |
| Time to death (median (IQR)) days | 87(40, 100) | 59 (20, 109) | 47 (27, 88) | 42 (15, 74) | 46 (18, 83) | 0.04 |
| Youth |  |  |  |  |  |  |
| Youths (15 to 35 years) | 1.054 (40) | 948 (42) | 886 (39) | 754 (40) | 698 (41) | 0.98 |
| Youths mortality | 33 (3.1) | 26 (2.7) | 27 (3.0) | 22 (2.9) | 24 (3.4) | 0.76 |
| Type of patient |  |  |  |  |  |  |
| TB existing cases | 220 (8.4) | 205 (9.0) | 219 (9.6) | 215 (11.5) | 150 (8.9) | 0.05 |
| TB existing cases mortality | 12 (5.5) | 17 (8.3) | 13 (5.9) | 19 (8.8) | 12 (8.0) | 0.58 |
| TB diagnosis |  |  |  |  |  |  |
| Sputum positive | 936 (36) | 908 (40) | 847 (37) | 991 (53) | 788 (47) | 0.37 |
| GeneXpert positive | 0 | 3 (0.1) | 9 (0.4) | 18 (1.0) | 133 (7.9) | 0.003 |
| Extra-pulmonary TB | 301 (12) | 265 (12) | 274 (12) | 232 (12) | 232 (14) | 0.08 |
| Clinical diagnosed | 1373 (53) | 1098 (48) | 1141 (50) | 632 (34) | 536 (34) | 0.008 |
| TB diagnosis deaths |  |  |  |  |  |  |
| Sputum positive | 24 (2.5) | 23 (2.9) | 29 (3.4) | 37 (3.7) | 47 (6.0) | 0.02 |
| GeneXpert positive | 0 | 0 | 0 | 3 (17) | 6 (4.5) | - |
| Extra-pulmonary TB | 15 (5.0) | 16 (6.0) | 23 (8.4) | 16 (6.9) | 24 (10) | 0.002 |
| Clinical diagnosed | 54 (3.9) | 76 (6.9) | 85 (7.4) | 54 (8.5) | 53 (9.9) | 0.004 |
| HIV status |  |  |  |  |  |  |
| HIV infected | 729 (28) | 659 (29) | 735 (32) | 529 (28) | 511 (30) | 0.89 |
| HIV infected mortality rate per 100PY | 15.87 | 21.72 | 25.95 | 27.78 | 31.77 | 0.001 |

PY-person years at risk
