# Supplementary material for: Mortality during treatment for tuberculosis; a review of surveillance data in a rural county in Kenya
Source: PLoS One. 2019 Jul 11;14(7):e0219191. doi: 10.1371/journal.pone.0219191 (PMC6622488; doi:10.1371/journal.pone.0219191)
Supplement: S2 Table — (DOCX) [file pone.0219191.s005.docx]

**S2 Table:** Univariable associations between features at initiating TB treatment and deaths within six months of follow up.

| **Features** | **Deaths (N=585)** | **Mortalityrate per 100PY (95% CI)** | **Crude**  **SHR (95% CI)** | **P-value** |
| --- | --- | --- | --- | --- |
| Age in years |  |  |  |  |
| <15 years | 33 (2.6) | 5.69 (3.51, 8.78) | 0.26 (0.20, 0.34) | <0.0001 |
| 15 to 45 years | 274 (4.2) | 9.26 (6.78, 12.74) | 0.41 (0.6, 0.47) | <0.0001 |
| 45 and above years | 278 (9.7) | 22.32 (16.53, 30.77) | Reference |  |
| Sex |  |  |  |  |
| Male | 338 (5.5) | 12.31 (8.58, 17.62) | Reference |  |
| Female | 247 (5.5) | 12.12 (8.66, 17.15) | 0.99 (0.93, 1.07) | 0.97 |
| Patient type |  |  |  |  |
| New cases | 512 (5.3) | 11.79 (8.08, 17.32) | Reference |  |
| Re-treatment cases | 73 (7.2) | 16.47 (13.13, 20.40) | 1.37 (0.99, 1.90) | 0.05 |
| TB type |  |  |  |  |
| Pulmonary | 491 (5.2) | 11.7 (8.2-16.6) | Reference |  |
| Extrapulmonary | 94 (7.2) | 16.4 (11.5-24.0) | 1.40 (1.17-1.67) | <0.001 |
| Type of health facility |  |  |  |  |
| Public | 427 (5.1) | 11.38 (8.47, 15.10) | Reference |  |
| Private | 155 (7.1) | 16.34 (7.33, 48.80) | 1.42 (0.83, 2.43) | 0.21 |
| Prisons | 3 (1.6) | 3.58 (2.74, 5.26) | 0.32 (0.24, 0.42) | <0.0001 |
| DOT |  |  |  |  |
| Family-based | 506 (5.5) | 12.21 (8.43, 18.07) | Reference |  |
| Community volunteer | 33 (4.1) | 9.20 (8.07, 11.89) | 0.75 (0.50, 1.14) | 0.18 |
| Health worker | 46 (7.2) | 16.30 (11.89, 21.94) | 1.32 (0.95, 1.84) | 0.09 |
| Nutrition status |  |  |  |  |
| Undernourished | 305 (6.3) | 14.20 (12.68,15.88) | 1.56 (1.48, 1.64) | <0.0001 |
| Normal | 151 (4.1) | 8.97 (7.65, 10.52) | Reference |  |
| Overweight | 41 (4.9) | 11.09 (8.17, 15.07) | 1.22 (0.69, 2.13) | 0.50 |
| Missing anthropometrics | 88 (6.6) | 15.07 (12.23, 18.58) | 1.63 (1.10, 2.40) | 0.01 |
| HIV status |  |  |  |  |
| HIV uninfected | 251 (3.4) | 7.46 (4.75, 11.63) | Reference |  |
| HIV infceted on ARVS | 292 (9.9) | 22.72 (17.52, 29.48) | 3.01 (2.34, 3.87) | <0.0001 |
| HIV infected not on ARVS | 33 (17) | 42.36 (31.91, 54.44) | 5.30 (2.79, 10.06) | <0.0001 |
| Unknown HIV status | 9 (6.4) | 15.67 (6.18, 54.11) | 1.93 (0.95, 3.93) | 0.07 |
| Treatment regimen |  |  |  |  |
| 2RHZE/4RH | 491 (5.2) | 11.70 (8.23, 16.67) | Reference |  |
| 2SRHZE/1RHZE/5RHE | 85 (7.9) | 18.05 (13.84, 24.37) | 1.51 (1.21, 1.89) | <0.0001 |
| 2RHZ/4RH | 7 (3.0) | 6.62 (2.84, 14.10) | 0.57 (0.29, 1.10) | 0.09 |
| Others | 2 (8.0) | 16.58 (5.10, 75.55) | 1.49 (0.47, 4.73) | 0.50 |
| Year of diagnosis |  |  |  |  |
| 2012 | 93 (3.6) | 7.79 (4.34, 14.01) | Reference |  |
| 2013 | 115 (5.1) | 11.37 (6.75, 20.08) | 1.44 (1.17, 1.78) | 0.001 |
| 2014 | 137 (6.0) | 13.45 (8.98, 20.46) | 1.73 (1.30, 2.29) | <0.0001 |
| 2015 | 110 (5.9) | 13.30 (9.98, 17.96) | 1.68 (1.09, 2.58) | 0.02 |
| 2016 | 130 (7.7) | 17.73 (14.40, 21.84) | 2.22 (1.33, 3.70) | 0.002 |

SHR-Subdistribution hazard ratios, DOT-directly observed treatment
