# Supplementary material for: Mortality during treatment for tuberculosis; a review of surveillance data in a rural county in Kenya
Source: PLoS One. 2019 Jul 11;14(7):e0219191. doi: 10.1371/journal.pone.0219191 (PMC6622488; doi:10.1371/journal.pone.0219191)
Supplement: S3 Table — (DOCX) [file pone.0219191.s006.docx]

**S3 Table:** Associations between features at initiating TB treatment and deaths within six months of follow-up among confirmed TB cases.

| **Features** | **Deaths (N=169)** | **Mortalityrate per 100PY (95% CI)** | **Adjusted**  **SHR (95% CI)** | **P-value** |
| --- | --- | --- | --- | --- |
| Age in years |  |  |  |  |
| <15 years | 2 (2.4) | 5.1 (1.3-20.5) | 0.34 (0.11-1.09) | 0.07 |
| 15 to 45 years | 104 (3.0) | 6.6 (5.4-8.0) | 0.49 (0.34-0.69) | <0.0001 |
| 45 and above years | 63 (6.0) | 13.1 (10.3-16.8) | Reference |  |
| Nutrition status |  |  |  |  |
| Undernourished | 96 (4.3) | 9.6 (7.8-11.7) | 1.63 (1.32-2.01) | <0.0001 |
| Normal | 41 (2.5) | 5.3 (3.9-7.2) | Reference |  |
| Overweight | 11 (3.5) | 7.8 (4.3-14.1) | 1.54 (1.04-2.29) | 0.03 |
| Missing anthropometrics | 21 (5.2) | 11.7 (7.6-17.9) | 2.02 (1.09-3.74) | 0.03 |
| HIV status |  |  |  |  |
| HIV uninfected | 64 (1.9) | 4.1 (3.2)-5.2 | Reference |  |
| HIV infected on ARVS | 91 (8.4) | 19.2 (15.6-23.6) | 4.57 (3.88-5.39) | <0.0001 |
| HIV infected not on ARVS | 12 (16) | 40.3 (22.9-70.9) | 11.08 (5.14-23.89) | <0.0001 |
| Unknown HIV status | 2 (4.0) | 9.5 (2.4-38.1) | 2.24 (0.42-11.86) | 0.34 |
| Year of diagnosis |  |  |  |  |
| 2012 | 24 (2.6) | 5.7 (3.8-8.4) | Reference |  |
| 2013 | 23 (2.5) | 5.5 (3.7-8.3) | 0.93 (0.78-1.11) | 0.43 |
| 2014 | 29 (3.4) | 7.2 (5.0-10.4) | 1.28 (0.61-2.67) | 0.51 |
| 2015 | 40 (4.0) | 8.9 (6.5-12.1) | 1.72 (0.96-3.10) | 0.07 |
| 2016 | 53 (5.8) | 13.2 (10.1-17.2) | 2.44 (1.25-4.75) | 0.009 |
| Model Performance |  |  |  |  |
| AUC (95% CI) |  |  | 0·77 (0·73-0·80) | |
| Bootstrapped AUC (95% CI) |  |  | 0.75 (0.71-0.80) | |

SHR-Sub-distribution hazard ratios, PY-person year, AUC-Area under receiver operating curve, TB cases confirmed by either positive sputum or GeneXpert test, The SHR are obtained using Fine and Gray competing risk regression analysis.
