# Supplementary material for: Mortality during treatment for tuberculosis; a review of surveillance data in a rural county in Kenya
Source: PLoS One. 2019 Jul 11;14(7):e0219191. doi: 10.1371/journal.pone.0219191 (PMC6622488; doi:10.1371/journal.pone.0219191)
Supplement: S4 Table — (DOCX) [file pone.0219191.s007.docx]

**S4 Table:** Associations between features at initiating TB treatment and deaths stratified by deaths within and after three months of starting TB treatment.

|  | **Deaths <3 months after starting TB treatment** | | **Deaths after 3 months of starting TB treatment** | |
| --- | --- | --- | --- | --- |
|  | **Adjusted**  **SHR (95% CI)** | **P-value** | **Adjusted**  **SHR (95% CI)** | **P-value** |
| Age in years |  |  |  |  |
| <15 years | 0.39 (0.34-0.45) | <0.001 | 0.47 (0.30-0.74) | 0.001 |
| 15 to 45 years | 0.30 (0.21-0.42) | <0.001 | 0.39 (0.21-0.74) | 0.004 |
| 45 and above years | Reference |  | Reference |  |
| TB diagnosis |  |  |  |  |
| Sputum positive | Reference |  | Reference |  |
| GeneXpert positive | 0.83 (0.43-1.60) | 0.58 | 0.74 (0.07-8.25) | 0.81 |
| Extrapulmonary TB | 1.41 (1.01-1.98) | 0.05 | 2.49 (1.74-3.57) | <0.001 |
| Clinically confirmed | 1.49 (1.25-1.77) | <0.001 | 2.30 (1.50-3.55) | <0.001 |
| HIV status |  |  |  |  |
| HIV uninfected | Reference |  | Reference |  |
| HIV infected on ARVS | 2.50 (2.09-3.00) | <0.001 | 3.32 (2.47-4.46) | <0.001 |
| HIV infected not on ARVS | 3.19 (2.05-4.97) | <0.001 | 3.64 (1.98-6.69) | <0.001 |
| Unknown HIV status | 0.94 (0.42-2.11) | 0.88 | - |  |
| Treatment regimen |  |  |  |  |
| 2RHZE/4RH |  |  | Reference |  |
| 2SRHZE/1RHZE/5RHE |  |  | 1.58 (1.05-2.36) | 0.03 |
| 2RHZ/4RH |  |  | 0.51 (0.05-5.53) | 0.58 |
| Others |  |  | 4.85 (1.64-14.37) | 0.004 |
| DOT |  |  |  |  |
| Family-based | Reference |  |  |  |
| Community volunteer | 0.98 (0.64-1.48) | 0.91 |  |  |
| Health worker | 0.79 (0.63-0.99) | 0.04 |  |  |

SHR-Sub-distribution hazard ratios, PY-person year, DOT-directly observed treatment, The SHR are obtained using Fine and Gray competing risk regression analysis.
